# Prolonged early postoperative adduction restriction is significantly associated with shoulder stiffness 1 year after arthroscopic rotator cuff repair

**DOI:** 10.1016/j.jseint.2025.05.006

**Published:** 2025-05-28

**Authors:** Yuki Miyasaka, Takahiro Sekiguchi, Norimasa Takahashi, Toshiki Nagaoka, Keisuke Kimura, Kohei Matsuda, Keisuke Matsuki, Shota Hoshika, Hiroyuki Sugaya, Itaru Kawashima

**Affiliations:** a Department of Rehabilitation, Funabashi Orthopaedic Clinic, Funabashi, Chiba, Japan; b Sports Medicine and Joint Center, Funabashi Orthopaedic Hospital, Funabashi, Chiba, Japan; c Medical Corporation Tanaka Clinic, Chomeigaoka, Miyagi, Japan; d Takeda Orthopaedic Clinic, Koriyama, Fukushima, Japan; e Tamura Orthopedics Clinic, Shizuoka-si, Shizuoka, Japan; f Tokyo Sports & Orthopaedic Clinic, Tokyo, Japan; g Department of Orthopaedic Surgery and Sports Medicine, University of Florida, Gainesville, FL, USA; h Department of Orthopaedic Surgery, Yachiyo Hospital, Anjo, Aichi, Japan

**Keywords:** Rotator cuff tear, Stiffness, Arthroscopic rotator cuff repair, Adduction restriction, Range of motion, Rehabilitation

## Abstract

**Background:**

Postoperative shoulder stiffness (POSS) is one of the most common complications of arthroscopic rotator cuff repair (ARCR). Although adduction restriction is commonly observed in the early postoperative period, it remains unclear whether a prolonged duration of this restriction impacts the development of POSS. The aim of this study was to investigate whether early postoperative adduction restriction is associated with the occurrence of POSS 1 year after ARCR.

**Methods:**

This retrospective cohort study included patients who underwent ARCR at a single institution between April 2017 and June 2018. A total of 96 shoulders were analyzed. Active and passive range of motion were assessed preoperatively and at 12 months postoperatively. Adduction restriction was evaluated in two positions: the droop position and the intermediate position. Adduction restriction was defined as resolved when the medial side of the arm made contact with the side of the body. Based on these evaluations, the duration of persistent postoperative adduction restriction was evaluated. POSS was defined as passive external rotation at the side of less than 30° at 12 months after surgery according to the previous studies. Patients were assigned to one of two groups: Group N, without POSS group, or Group S, with POSS group. Propensity score matching was used to ensure comparability between groups. The duration of persistent postoperative adduction restriction was compared between the groups.

**Results:**

After propensity score matching, 38 shoulders were analyzed (19 in Group N and 19 in Group S). While no significant difference in the duration of persistent postoperative adduction restriction in the droop position between the groups was observed, the duration in the intermediate position was significantly shorter in Group N compared to Group S (7 ± 3 weeks vs. 10 ± 4 weeks, *P* = .007).

**Conclusion:**

The duration of persistent adduction restriction in the intermediate position was significantly longer in shoulders with POSS at 12 months after ARCR compared to those without POSS. Prolonged early postoperative adduction restriction may associate with the occurrence of POSS 12 months after ARCR.

Arthroscopic rotator cuff repair (ARCR) has been widely performed for rotator cuff tears, yielding highly favorable clinical outcomes.^26^ However, postoperative shoulder stiffness (POSS) is one of the most common complications of ARCR, often resulting in residual pain and reduced patient satisfaction.^1^^,^^14^^,^^20^ Various factors have been reported to be associated with POSS, including age, sex, rotator cuff tear size, preoperative range of motion (ROM), rehabilitation protocols, and the presence of diabetes mellitus.^2^, ^3^, ^4^^,^^6^^,^^22^^,^^23^,^27^ Nevertheless, the causes of POSS have not yet been fully understood.

Adduction restriction has been reported to correlate with ROM loss in shoulders with rotator cuff tears.^28^ In addition, the adduction manipulation of the glenohumeral joint has been shown to restore ROM in such cases.^12^ Based on these findings, it is plausible that adduction restriction following ARCR may also be associated with POSS; however, this hypothesis remains uncertain. Furthermore, in our clinical practice, adduction restriction is commonly observed during the early postoperative period, and shoulders with a prolonged duration of early adduction restriction appear to be more frequently associated with POSS. Nevertheless, the potential contribution of this restriction to the development of POSS at 12 months postoperatively remains unclear.

The aim of this study was to investigate whether early postoperative adduction restriction is associated with the occurrence of POSS 1 year after ARCR. We hypothesized that shoulders with POSS at 12 months post-ARCR would have a significantly longer duration of early adduction restriction.

## Materials and methods

### Patients

This retrospective cohort study was approved by the Institutional Review Board and Ethics Committee of our institution. Patients who underwent ARCR at a single sports medicine center specializing in shoulder surgery between April 2017 and June 2018 and agreed to participate in this study were included. The exclusion criteria were as follows: (1) isolated subscapularis tendon repair, (2) massive/irreparable rotator cuff tears, (3) a follow-up duration of less than 12 months, and (4) incomplete adduction data. Shoulders with massive or irreparable rotator cuff tears were excluded, as these cases were treated with arthroscopic superior capsular reconstruction. Patient records, including surgical reports, were reviewed for preoperative demographic information (diagnosis, sex, age at surgery, body mass index, operative side, and workers compensation) and for rotator cuff tear size, categorized as incomplete tear, small tear (less than 1 cm in length), medium tear (1-3 cm), or large tear (3-5 cm).^5^ For the assessment of pain, nighttime rest pain was evaluated using the Numerical Rating Scale (NRS), with scores ranging from 0 (no pain) to 10 (unbearable pain). The NRS was assessed preoperatively and at 12 months postoperatively.

### Surgical procedure

Patients underwent surgery in the beach-chair position under general anesthesia with an interscalene block. All operations were performed either by a senior, experienced shoulder surgeon, or by a fellow surgeon under the supervision of a senior, experienced shoulder surgeon, using the same surgical technique. After the glenohumeral joint and the subacromial bursa were investigated arthroscopically, subacromial decompression was performed in all cases.

For shoulders with poor tendon mobility, coracohumeral ligament resection and capsular release were performed. Tendons with partial-thickness tears were made into full-thickness tears and repaired as such. Triple-loaded suture anchors (HEALIX ADVANCE BR Anchor; DePuy Synthes, Raynham, MA, USA) were inserted at the medial border of the greater tuberosity, and 2 suture limbs at a time were passed through the cuff using a suture grasper. Two pairs of suture limbs were fixed using lateral-row knotless suture anchors (HEALIX ADVANCE Knotless Anchor, Depuy Synthes, Raynham, MA, USA; and SwiveLock C Anchor, Arthrex, Naples, FL, USA). Then, the remaining sutures were tied to avoid excessive stress concentration on the medial-row sutures.^25^ We usually use 1 or 2 anchors each for the medial and lateral rows in smaller tears and 2 or 3 anchors each in larger tears. A suture-bridge technique was also utilized for the repair of concomitant subscapularis tears.^24^ All patients underwent arthroscopic subacromial decompression. Tenodesis or tenotomy of the long head of the biceps was performed in shoulders with rotator cuff tears larger than medium in size or with long head of the biceps inflammation or laceration.^13^ Generally, we performed tenodesis for male patients younger than 70 years and female patients younger than 65 years of age.

### Postoperative rehabilitation

The shoulders were immobilized for 4 or 5 weeks using a sling immobilizer with an abduction pillow. Isometric rotator cuff exercises and relaxation techniques for the shoulder girdle muscles were initiated on the day following surgery. After the immobilization period, passive and active-assisted exercises focusing on forward flexion and external rotation were introduced, ensuring that pain was not provoked. At 6 weeks post-surgery, patients began strengthening exercises targeting the rotator cuff and scapular stabilizers. Patients typically attended physiotherapy sessions once or twice a week and performed home exercises on the remaining days. Light sports activities were permitted 3 months post-surgery, with a gradual return to full, unrestricted activities by 6 months, depending on the patient's functional recovery.^9^^,^^16^^,^^24^, ^25^, ^26^

### Assessment of range of motion

ROM was assessed preoperatively and at 12 months postoperatively. Active and passive forward elevation, abduction, and external rotation were measured using a hand-held goniometer. Active internal rotation was assessed with a tape measure, recording the distance (in centimeters) from the spinous process of the seventh cervical vertebra to the thumb. Passive external rotation at the side, measured 12 months postoperatively, was used as a criterion for POSS, based on previous studies.^4^^,^^18^ POSS was defined as passive external rotation at the side of less than 30° at 12 months after surgery.^4^^,^^18^ Thus, patients were assigned to one of the two groups: Group N, without POSS group, or Group S, with POSS group.

### Assessment of adduction restriction

**Figure 1:**
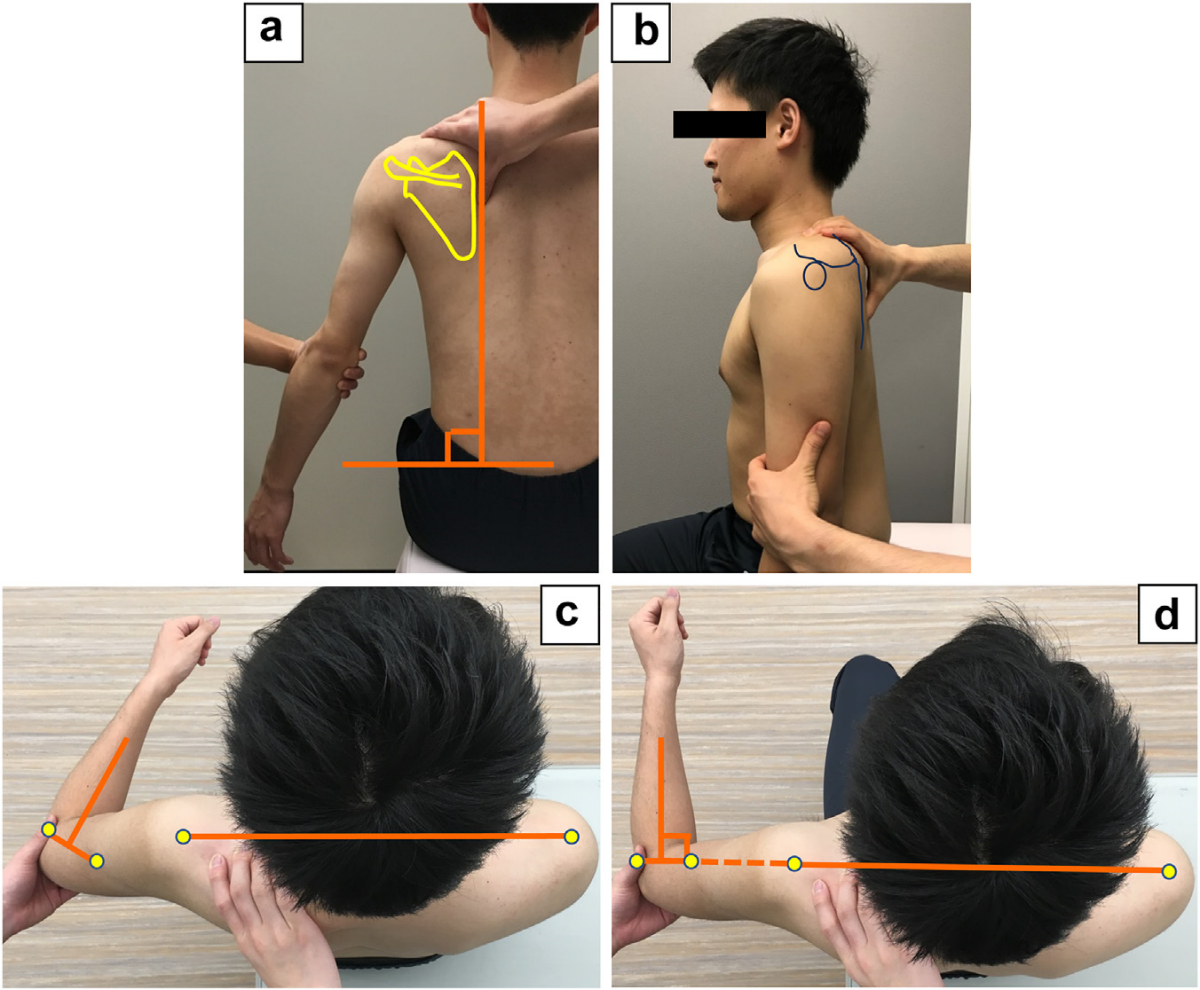
The method for assessing adduction restriction. (**a**) The medial border of the scapula was fixed to remain perpendicular to the floor, (**b**) the medial border of the scapula was fixed to ensure that the scapula did not tilt anteriorly and downwardly from its starting position, (**c**) the assessment of adduction restriction in the droop position. Patients were instructed to relax and adduct the shoulder, and (**d**) the assessment of adduction restriction in the intermediate position. Patients were instructed to flex the elbow to 90° and align the line passing through the lateral and medial epicondyles with the coronal plane.

The assessment of adduction restriction was performed in two positions: the droop position and the intermediate position, which have been previously reported as highly reliable methods: droop position (intraclass correlation coefficients [ICC] ICC1.1: 0.97, ICC2.1: 0.90) and intermediate position (ICC1.1: 0.97, ICC2.1: 0.79).^15^ Measurements were conducted in both positions to mitigate the potential underestimation of adduction restriction, which may occur due to compensatory internal rotation of the shoulder joint when only the droop position is used.^15^ During both measurements, the scapula was stabilized to prevent compensatory movement. The medial border of the scapula was fixed to remain perpendicular to the floor and to ensure that the scapula did not tilt anteriorly and downwardly from its starting position (Fig. 1, *A* and *B*). In the droop position, patients were instructed to relax and adduct the shoulder (Fig. 1, *C*). In the intermediate position, patients were instructed to flex the elbow to 90° and align the line passing through the lateral and medial epicondyles with the coronal plane (Fig. 1, *D*). Subsequently, they extended the elbow and performed adduction. Adduction restriction was defined as resolved when the medial side of the arm contacted the side of the body.

Measurements were performed passively within a painless range by five experienced physical therapists. They had previously standardized their measurement methods before the start of the study. The assessment of adduction restriction was conducted weekly from the first postoperative week for up to 12 weeks, and the week of resolution was recorded. If adduction restriction was not resolved by 12 weeks, it was recorded as 13 weeks. Based on these assessments, the duration of persistent postoperative adduction restriction was evaluated.

### Statistical analysis

All statistical analyses were performed using EZR (Saitama Medical Center, Jichi Medical University, Saitama, Japan), which is a graphical user interface for R (The R Foundation for Statistical Computing, Vienna, Austria). More precisely, it is a modified version of R commander designed to add statistical functions frequently used in biostatistics.

To reduce potential bias between Group N and Group S, propensity score matching was employed. Propensity scores were estimated using a logistic regression model based on the following factors: age at surgery, sex, body mass index, operative side, tear size, workers compensation, and preoperative ROM (passive forward elevation and external rotation), and the presence of diabetes mellitus, which have been reported to be associated with POSS.^2^, ^3^, ^4^^,^^6^^,^^20^^,^^23^,^27^ Matching was performed in a one-to-one ratio with a caliper width of 0.2.

The Student's t-test and Fisher's exact test were performed to compare demographic and ROM data between the groups. The Student's t-test was also employed to compare the duration of persistent postoperative adduction restriction between the groups. *P* values < .05 were considered statistically significant. A post hoc power analysis was performed based on the difference in the duration of persistent postoperative adduction restriction in the intermediate position between the groups by use of an α value of 0.05.

## Results

**Figure 2:**
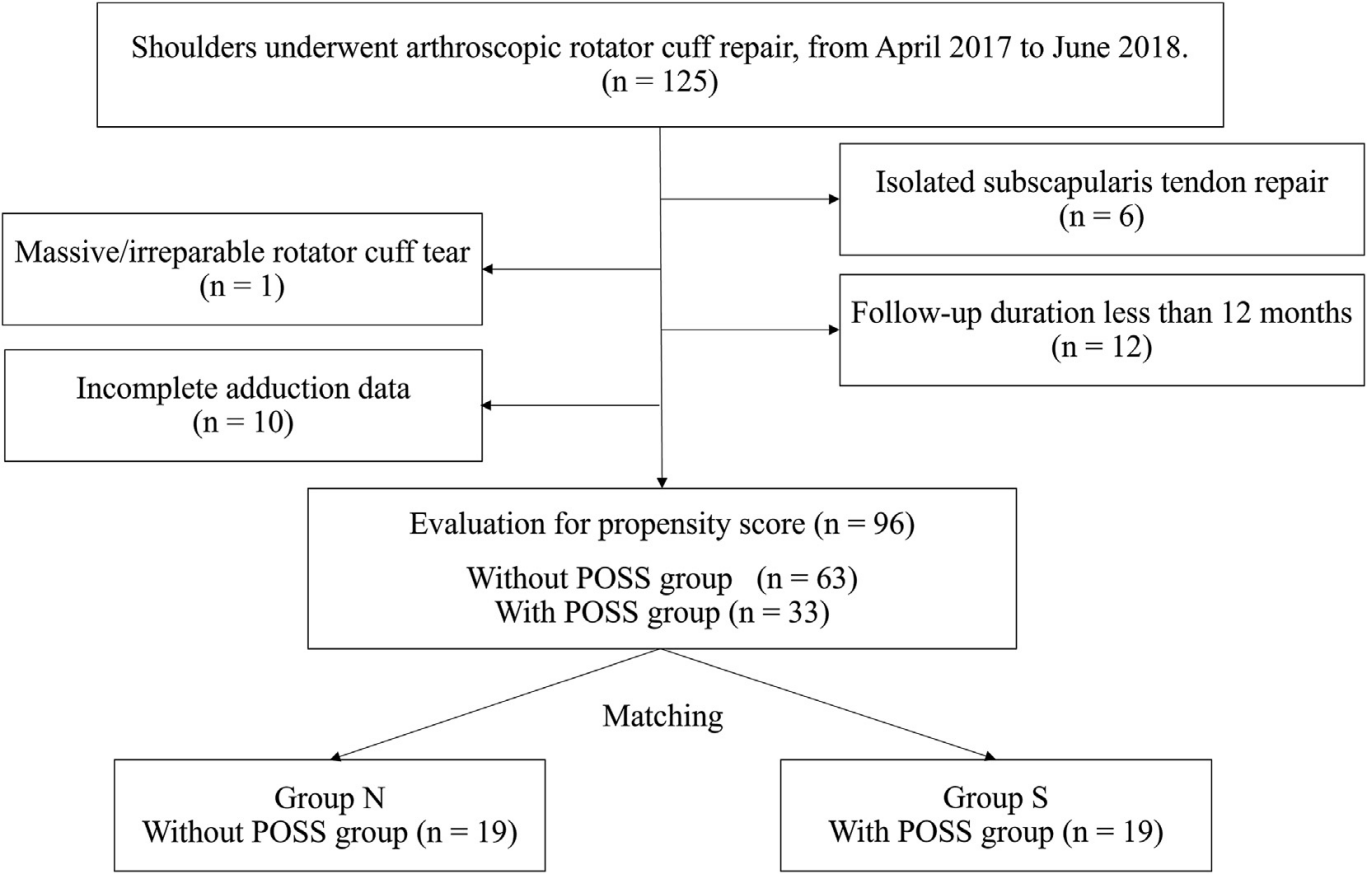
Flowchart of the patient selection. *POSS*, postoperative shoulder stiffness.

In total, 125 shoulders that underwent ARCR between April 2017 and June 2018 were identified. Of these, 29 shoulders were excluded due to isolated subscapularis tendon repair (n = 6), massive/irreparable rotator cuff tear (n = 1), follow-up duration of less than 12 months (n = 12), and incomplete adduction data (n = 10) (Fig. 2).

Among the remaining 96 shoulders, 63 were classified into Group N and 33 were classified into Group S. Significant differences were observed in sex, the presence of diabetes, rotator cuff tear size, preoperative active external/internal rotation, and preoperative passive external rotation before propensity score matching (Table I). After propensity score matching, 38 shoulders were included in the analysis, with 19 in Group N and 19 in Group S. The demographic and preoperative clinical characteristics were similar between the groups (Table II).

**Table I:** Demographic data and preoperative range of motion prior to propensity score matching.

	Group N n = 63	Group S n = 33	*P* value
Sex, Male / Female	35/28	26/7	**.028**
Age at surgery, year	62 ± 11	66 ± 10	.086
BMI, kg/m^2^	24.1 ± 2.7	24.1 ± 3.2	.973
Operative side, Right/Left	44/19	20/13	.373
Diabetes mellitus, Yes/No	4/59	7/26	**.043**
Workers compensation, Yes/No	0/63	2/31	.116
Rotator cuff tear size, Partial/Small/Medium/large	15/18/22/8	5/7/9/12	**.031**
Preoperative nighttime rest pain using NRS	4.9 ± 2.0	5.0 ± 2.5	.735
Preoperative active ROM			
Abduction, degree	109 ± 43	107 ± 38	.822
Forward elevation, degree	124 ± 36	123 ± 30	.887
External rotation, degree	40 ± 15	22 ± 16	**<.001**
Internal rotation, cm	30 ± 10	36 ± 13	**.013**
Preoperative passive ROM			
Abduction, degree	122 ± 39	117 ± 34	.548
Forward elevation, degree	138 ± 29	135 ± 29	.542
External rotation, degree	47 ± 15	29 ± 15	**<.001**

*BMI*, body mass index; *ROM*, range of motion; *NRS*, numeric rating scale.

The values are given as mean ± standard deviation.

Bold text indicates a significant *P* value.

**Table II:** Demographic data and preoperative ROM of the matched patients.

	Group N n = 19	Group S n = 19	*P* value
Sex, Male/Female	12/7	13/6	1.000
Age at surgery, year	62 ± 14	63 ± 11	.839
BMI, kg/m^2^	24.6 ± 2.8	24.7 ± 3.7	.937
Operative side, Right/Left	11/8	13/6	.737
Diabetes mellitus, Yes/No	3/16	3/16	1.000
Workers compensation, Yes/No	0/19	0/19	1.000
Rotator cuff tear size, Partial/Small/Medium/large	4/6/5/4	3/6/6/4	.758
Preoperative nighttime rest pain using NRS	5.3 ± 2.0	5.6 ± 2.4	.705
Preoperative active ROM			
Abduction, degree	106 ± 40	108 ± 35	.878
Forward elevation, degree	123 ± 34	121 ± 29	.843
External rotation, degree	27 ± 15	28 ± 15	.983
Internal rotation, cm	30 ± 15	38 ± 14	.069
Preoperative passive ROM			
Abduction, degree	115 ± 37	116 ± 34	.931
Forward elevation, degree	135 ± 30	132 ± 27	.761
External rotation, degree	34 ± 14	34 ± 15	.991

*BMI*, body mass index; *ROM*, range of motion; *NRS*, numeric rating scale.

The values are given as mean ± standard deviation.

The NRS scores for nighttime rest pain at 12 months in Group N were also significantly lower than those in Group S (1.2 ± 1.8 vs. 3.1 ± 2.4, *P* = .018). Regarding postoperative ROM, active abduction, forward elevation, and external rotation angles in Group N were significantly greater than those in Group S (155 ± 18° vs. 136 ± 28°, *P* = .019, 153 ± 12° vs. 141 ± 17°, *P* = .024, and 42 ± 6° vs. 15 ± 8°, *P* < .001, respectively) (Table III). Postoperative passive abduction, forward elevation, and external rotation angles in Group N were also significantly greater than those in Group S (161 ± 18° vs. 140 ± 28°, *P* = .011, 160 ± 12° vs. 147 ± 18°, *P* = .015, and 48 ± 8° vs. 20 ± 8°, *P* < .001, respectively).

**Table III:** ROM at 12 months postoperatively.

	Group N n = 19	Group S n = 19	*P* value
Postoperative active ROM			
Abduction, degree	155 ± 18	136 ± 28	**.019**
Forward elevation, degree	153 ± 12	141 ± 17	**.024**
External rotation, degree	42 ± 6	15 ± 8	**<.001**
Internal rotation, cm	26 ± 8	32 ± 11	.061
Postoperative passive ROM			
Abduction, degree	161 ± 18	140 ± 28	**.011**
Forward elevation, degree	160 ± 12	147 ± 18	**.015**
External rotation, degree	48 ± 8	20 ± 8	**<.001**
Postoperative nighttime rest pain using NRS	1.2 ± 1.8	3.1 ± 2.4	**.018**

*ROM*, range of motion; *NRS*, numeric rating scale.

The values are given as mean ± standard deviation.

Bold text indicates a significant *P* value.

Internal rotation was assessed with a tape measure, recording the distance from the spinous process of the seventh cervical vertebra to the thumb.

No significant difference was observed in the duration of persistent postoperative adduction restriction in the droop position between the groups (Table IV). However, the duration of persistent postoperative adduction restriction in the intermediate position in Group N was significantly shorter than that in Group S (7 ± 3 weeks vs. 10 ± 4 weeks, *P* = .007). The post hoc power analysis indicated that the power was 0.872.

**Table IV:** The duration of persistent postoperative adduction restriction.

	Group N n = 19	Group S n = 19	*P* value
In droop position, week	5 ± 3	6 ± 4	.744
In intermediate position, week	7 ± 3	10 ± 4	**.007**

The values are given as mean ± standard deviation.

Bold text indicates a significant *P* value.

## Discussion

This study demonstrated that the duration of persistent adduction restriction in the intermediate position was significantly longer in shoulders with POSS at 12 months after ARCR compared to those without POSS. Prolonged early postoperative adduction restriction may be correlated with the occurrence of POSS.

As POSS adversely affects clinical outcomes and sometimes necessitates arthroscopic capsular release, it is crucial to identify its causes and address them appropriately.^1^^,^^3^^,^^4^^,^^14^^,^^20^ While factors such as age, sex, rotator cuff tear size, preoperative ROM, rehabilitation protocols, and the presence of diabetes have been implicated, the exact causes of POSS remain poorly understood due to the complex interplay of multifactorial influences.^2^, ^3^, ^4^^,^^6^^,^^21^^,^^25^ In the present study, significant differences were observed between the groups before propensity score matching in sex, rotator cuff tear size, preoperative ROM, and the presence of diabetes, consistent with previous reports. To isolate the direct relationship between adduction restriction and POSS, these variables were adjusted using propensity score matching.

The consequences and causes of adduction restriction in the shoulder joint are poorly understood. Specifically, early postoperative adduction restriction, commonly observed after ARCR in clinical practice, remains insufficiently studied and not well understood. Previous reports have demonstrated that adduction restriction in shoulders with rotator cuff tears is associated with ROM loss and that improvement in adduction restriction leads to restored ROM.^12^^,^^28^ Based on these findings, this study was conducted. The present study demonstrated that shoulders with POSS at 12 months had a longer duration of persistent adduction restriction in the intermediate position, consistent with our hypothesis. Prolonged early postoperative adduction restriction in the intermediate position can be considered to be significantly associated with the occurrence of POSS.

The glenohumeral joint tends to be more internally rotated in the drooping position compared to the intermediate position, and achieving adduction in the intermediate position requires greater external rotation than in the drooping position.^15^ The ability to achieve external rotation is influenced by the flexibility and function of soft tissues located in the anterosuperior part of the glenohumeral joint, such as the coracohumeral ligament and the supraspinatus muscle.^8^^,^^11^^,^^17^^,^^19^ Furthermore, the coracohumeral ligament has been reported to be stretched during passive external rotation or adducted positions.^11^ Based on findings from previous studies and our study—which indicated that a prolonged duration of persistent adduction restriction in the intermediate position was associated with the occurrence of POSS—it may be suggested that early postoperative adduction restriction in the intermediate position is related to increased stiffness of the coracohumeral ligament and reduced flexibility or function of the supraspinatus muscle. If this condition persists during the early postoperative period, it could hinder ROM improvement even 12 months after ARCR and potentially associate with the development of POSS.

Another notable finding in our study was the absence of a significant difference in the duration of adduction restriction in the droop position between groups, despite a significant difference being observed in the intermediate position. It has been reported that adduction restriction in the droop position, a measurement commonly used in clinical settings, may be underestimated due to compensatory internal rotation of the shoulder.^15^ This underestimation may be more likely in scenarios where external rotation restriction is prevalent, particularly in the early period after ARCR. The findings of the present study suggest that the intermediate position may be more sensitive in detecting early adduction restriction, which is clinically relevant to the development of POSS.

It has been reported that postoperative pain is associated with POSS.^6^ Moreover, pain is closely related to limited shoulder motion.^10^ In the present study, shoulders with POSS also showed significantly higher rest pain at night 1 year after surgery. This difference in pain might be related to early postoperative adduction restriction, or external rotation may have contributed to increased pain. Consequently, careful pain management may be required for shoulders with early postoperative adduction restriction.

Based on our findings, it might be desirable to initiate intervention in the early postoperative period to resolve adduction restriction in the intermediate position at an earlier stage after ARCR. However, there is a concern that earlier resolution of adduction restriction may increase the load on the rotator cuff after repair.^7^ Future studies should explore whether interventions specifically targeting adduction restriction in the intermediate position, such as focused stretching, manual therapy, or novel rehabilitation techniques, can effectively reduce the risk of POSS with or without increasing the risk of failure after ARCR.

This study has several limitations. First, it was conducted at a single center, which may limit the generalizability of the findings. Second, although propensity score matching was employed to minimize confounding factors, residual confounding might not be entirely excluded as the cause of POSS has not been entirely certain. Third, this study did not evaluate other potential factors, such as preoperative adduction restriction and psychological factors, which might also contribute to POSS. Fourth, we did not evaluate muscle strength and magnetic resonance imaging findings; therefore, it is unclear whether POSS affects there. Fifth, a detailed assessment of glycemic control was not possible in this study, as diabetes management was performed at outside institutions. Finally, this study did not investigate whether improving adduction restriction by a specific intervention during the early postoperative period could actually reduce the occurrence of POSS. Future prospective studies are needed to address this issue.

## Conclusion

This study demonstrated that the duration of persistent adduction restriction in the intermediate position was significantly longer in shoulders with POSS at 12 months after ARCR compared to those without POSS. Prolonged early postoperative adduction restriction may associate with the occurrence of POSS 12 months after ARCR.

## Disclaimers:

Funding: No funding was disclosed by the authors.

Conflicts of interest: Norimasa Takahashi has received speaker honoraria from DePuy Synthes, Medacta International outside of the submitted work. Hiroyuki Sugaya has received speaker honoraria from DePuy Synthes, Smith & Nephew, Zimmer, Biomet, and Stryker outside of the submitted work. The other authors, their immediate families, and any research foundation with which they are affiliated have not received any financial payments or other benefits from any commercial entity related to the subject of this article.
